# Granulocyte colony-stimulating factor attenuates myocardial remodeling and ventricular arrhythmia susceptibility via the JAK2-STAT3 pathway in a rabbit model of coronary microembolization

**DOI:** 10.1186/s12872-020-01385-5

**Published:** 2020-02-17

**Authors:** Weiwei Wang, Shuhua Ye, Lutao Zhang, Qiong Jiang, Jianhua Chen, Xuehai Chen, Feilong Zhang, Hangzhou Wu

**Affiliations:** 1 Department of Cardiology, Fujian Medical University Union Hospital, Fuzhou, 350001 China; 2 Department of Cardiology, Fujian Provincial People’s Hospital, Fuzhou, 350004 China; 3 Department of Cardiology, People’s Hospital of Wuqing District, Tianjin, 301700 China; 4 Fujian Medical University Union clinical medical college, Fuzhou, 350001 China

**Keywords:** G-CSF, JAK2-STAT3 signaling pathway, Microembolism, Ventricular arrhythmia, Cx43

## Abstract

**Background:**

Coronary microembolization (CME) has a poor prognosis, with ventricular arrhythmia being the most serious consequence. Understanding the underlying mechanisms could improve its management. We investigated the effects of granulocyte colony-stimulating factor (G-CSF) on connexin-43 (Cx43) expression and ventricular arrhythmia susceptibility after CME.

**Methods:**

Forty male rabbits were randomized into four groups (*n* = 10 each): Sham, CME, G-CSF, and AG490 (a JAK2 selective inhibitor). Rabbits in the CME, G-CSF, and AG490 groups underwent left anterior descending (LAD) artery catheterization and CME. Animals in the G-CSF and AG490 groups received intraperitoneal injection of G-CSF and G-CSF + AG490, respectively. The ventricular structure was assessed by echocardiography. Ventricular electrical properties were analyzed using cardiac electrophysiology. The myocardial interstitial collagen content and morphologic characteristics were evaluated using Masson and hematoxylin-eosin staining, respectively.

**Results:**

Western blot and immunohistochemistry were employed to analyze the expressions of Cx43, G-CSF receptor (G-CSFR), JAK2, and STAT3. The ventricular effective refractory period (VERP), VERP dispersion, and inducibility and lethality of ventricular tachycardia/fibrillation were lower in the G-CSF than in the CME group (*P* < 0.01), indicating less severe myocardial damage and arrhythmias. The G-CSF group showed higher phosphorylated-Cx43 expression (*P* < 0.01 vs. CME). Those G-CSF-induced changes were reversed by A490, indicating the involvement of JAK2. G-CSFR, phosphorylated-JAK2, and phosphorylated-STAT3 protein levels were higher in the G-CSF group than in the AG490 (*P* < 0.01) and Sham (*P* < 0.05) groups.

**Conclusion:**

G-CSF might attenuate myocardial remodeling via JAK2-STAT3 signaling and thereby reduce ventricular arrhythmia susceptibility after CME.

## Background

Early percutaneous coronary intervention (PCI) can restore blood flow to ischemic myocardium, decrease infarct size, and reduce mortality and complications [1], but about 10–30% of patients exhibit no-reflow or slow-reflow phenomena after PCI, which seriously affects prognosis [2]. The main cause of no-reflow or slow-reflow after PCI is coronary microembolism (CME) [3], resulting in myocardial cell necrosis and apoptosis, ventricular remodeling, malignant arrhythmia, and cardiac failure [4]. No drugs or mechanical devices are currently available to prevent CME, and remedial measures after the occurrence of CME have only a limited impact on the development of arrhythmias and prognosis [5].

Connexin-43 (Cx43) is the major protein of gap junctions in the cardiac ventricles and is crucial for the synchronized contraction of the heart [6]. Remodeling of Cx43 and phosphorylated Cx43 (p-Cx43) distribution after ischemia is thought to cause increased anisotropy of electrical conduction and abnormal synchronization and coordination of electrical activity, leading to ventricular arrhythmia [7]. Whether Cx43 remodeling occurs after CME and increases the susceptibility to ventricular arrhythmias is not fully understood.

Granulocyte colony-stimulating factor (G-CSF) can reduce myocardial apoptosis and inflammation, inhibit ventricular remodeling, improve cardiac function, stabilize the myocardial electrophysiological characteristics, and reduce the incidence of ventricular arrhythmia after ischemia-reperfusion injury and MI [8]. G-CSF can reduce the incidence of ventricular arrhythmia after MI in rats by promoting Cx43 expression in the infarcted region margins [9]. Furthermore, G-CSF was reported to decrease the infarct size, stabilize the myocardial electrophysiology, and increase the threshold for ventricular fibrillation in a model of ischemia-reperfusion [10]. G-CSF activates numerous signaling pathways after binding to its receptor (G-CSFR), including the JAK2-STAT3 pathway. JAK2-STAT3 signaling can reduce the apoptosis of cardiomyocytes and endothelial cells and inhibit myocardial remodeling [11]. The activation of JAK2-STAT3 increases the expression of Cx43 in various cells and tissues [12, 13]. It remains unknown whether G-CSF can reduce the incidence of ventricular arrhythmia after CME and, if so, whether JAK2-STAT3 signaling is involved.

We hypothesized that G-CSF would attenuate the structural and electrical remodeling of the myocardium after CME and reduce the susceptibility to ventricular arrhythmia by regulating Cx43 phosphorylation and distribution via the JAK2-STAT3 pathway. Therefore, we investigated the effects of G-CSF and a JAK2-STAT3 inhibitor (AG490) on myocardial remodeling and arrhythmia susceptibility in a novel rabbit model of CME.

## Material and methods

### Laboratory animals and grouping

Forty male New Zealand white rabbits (3.0–3.5 kg) were purchased from Songlian Laboratory Animal Center, Shanghai, China (certificate no. 2007001103447). All animals were housed at a constant temperature of 24 °C with a humidity of ~ 50% with a 12-h/12-h light/dark cycle (artificial lighting), with food and water available ad libitum. After 1 week of adaptive feeding, the rabbits were randomly divided into four groups: CME, G-CSF, AG490, and Sham (*n* = 10 per group). All experimental procedures were approved by the National Laboratory Animal Management Regulations and Fujian Provincial Laboratory Animal Management Regulations.

### Establishment of the rabbit model of CME

Each rabbit was sedated with ketamine and xylazine (25 and 3.75 mg·kg^− 1^ im), intubated, and ventilated with supplemental oxygen (2–4%). During the procedure, the rabbits were anesthetized with continuous intravenous infusion of ketamine and xylazine (5 and 4.5 mg·kg^− 1^·h^− 1^). The right common carotid artery was exposed and punctured, a guidewire was inserted into the ascending aorta under X-ray fluoroscopy, and a catheter was introduced into the left coronary artery for non-selective coronary angiography. For rabbits in the CME, G-CSF, and AG490 groups, autologous thrombus particles (5 mg, prepared using 1 ml of ear vein blood from each rabbit, dried into a blood clot at 37 °C, ground, and sieved with a 38-μm screen mesh to make an autologous microthrombus) were injected via the catheter into the LAD artery to establish the CME model. For rabbits in the Sham group, an equal volume of normal saline was injected. The rabbits in the G-CSF group received subcutaneous injections of G-CSF (10 μg/kg/d) beginning at 2 h after surgery and then daily for 6 days [14]. The rabbits in the AG490 group received an intraperitoneal injection of AG490 (a specific JAK2 inhibitor, 0.25 mg/kg/d) 2 h after surgery and 30 min before each subcutaneous injection of G-CSF (10 μg/kg/d) for 6 days [15].

### Measurement of body weight and heart rate

Bodyweight and heart rate were measured before and 2 weeks after surgery using an electronic scale for animals and a multi-channel electrophysiology recorder, respectively.

### Echocardiography

Echocardiography was performed before and 2 weeks after surgery, as previously described [16]. Each rabbit was anesthetized with ketamine and xylazine (25 and 3.75 mg·kg^− 1^ im). Echocardiography was carried out using a Vivid 7 ultrasound instrument (GE Healthcare, Chicago, IL, USA) and an S12 transducer. The following parameters were recorded: parasternal left ventricular end-diastolic dimension (LVED, mm), left ventricular end-systolic dimension (LVES, mm), interventricular septum dimension (IVS, mm), left ventricle posterior wall dimension (LVPW, mm), fractional shortening (FS, %), and left ventricular ejection fraction (LVEF, %). The average of three independent measurements was used for the analysis.

### Electrophysiology

The electrophysiology study was performed using a modification of a method described previously [17]. Two weeks after surgery, each animal was anesthetized with ketamine and xylazine (25 and 3.75 mg·kg^− 1^ im) and buprenorphine (0.03 mg·kg^− 1^ sq), intubated, and ventilated with isoflurane (1–2%, Fi_O2_ 0.5). Heart rate, QRS interval, and QT interval (lead II) were recorded using a multi-channel electrophysiology recorder. The right jugular vein and carotid artery were identified and separated and heparinized. Then, 4-French, quad-polar electrode catheters were inserted under X-ray guidance into the right and left ventricles through the jugular vein and carotid artery, respectively. The catheters were connected to a multi-channel electrophysiology recorder, and stimuli were applied at twice the diastolic threshold with a pulse width of 2 ms. The evaluated parameters were ventricular effective refractory period (VERP), VERP dispersion, and occurrence of ventricular tachycardia (VT) or ventricular fibrillation (VF). VERP was defined as the shortest S1-S2 interval that elicited an electrogram response in the absence of absolute refractoriness. VERP dispersion was defined as the maximum difference between VERPs measured at the left and right ventricular apex as well as the mitral valve and tricuspid annulus. The presence/absence of VT/VF was determined after the application of S1S1 stimuli (40-ms duration) at the right ventricular apex for 10 s, repeated three times at intervals of 3 min.

### Preparation of heart specimens

After completion of the electrophysiology experiments, the animals were sacrificed with an overdose of pentobarbitone sodium (111 mg·kg^− 1^, i.v.), and the heart was excised and washed in 0.9% pre-cooled (4 °C) saline. A portion of the left ventricle containing the papillary muscles was fixed in 10% neutral formalin for 24 h, embedded in paraffin, and sectioned to produce 10 consecutive sections with a thickness of 3.5 μm.

### Hematoxylin-eosin (HE) staining

Paraffin-embedded sections were dewaxed using xylene, rehydrated in graded ethanol, and washed in water. The sections were stained with hematoxylin and eosin using standard techniques. The slides were washed in water, graded alcohol, and xylene. After mounting in resinene, 10 fields were randomly selected from each section and observed under an inverted biological microscope (DMI3000B, Leica, Wetzlar, Germany). The ratio of the microinfarcted area to the total area (%) was calculated using the Image-Pro Plus (IPP) 6.0 (Media Cybernetics, Rockville, MD, USA) image analysis software.

### Masson trichrome staining

Standard techniques were used to stain paraffin-embedded sections with Wiegert’s iron hematoxylin (10 min), plasma stain (acid fuchsin, Xylidine Ponceau, glacial acetic acid, and distilled water; 5 min), phosphomolybdic acid (5 min), and aniline blue (15 min). Collagen fibers are stained blue, cardiomyocytes are stained red, and nuclei are stained blue-black. Sections were observed under the DMI3000B microscope. IPP6.0 was used to calculate the ratio of the area of collagen in the ventricular interstitium (excluding collagen around blood vessels) to the total area.

### Immunohistochemistry

The expression levels of phosphorylated Cx43 (p-Cx43), total Cx43 (t-Cx43), and G-CSFR in the anterior ventricular wall were evaluated. The paraffin sections were dewaxed, blocked with goat serum, and incubated overnight at 4 °C with goat anti-p-Cx43 (1:300, SC-25165, Santa Cruz Biotechnology, Santa Cruz, CA, USA), anti-t-Cx43 (1:2500, AB0016, Sicgen), or anti-G-CSFR (1:200, SC-323899, Santa Cruz Biotechnology, Santa Cruz, CA, USA) primary antibody. The sections were incubated with mouse anti-goat secondary antibody labeled with horseradish peroxidase (HRP) (ZSGB Biotechnology Co., Ltd., Beijing, China) for 20 min at 37 °C. The sections were developed using streptavidin and diaminobenzidine. Negative controls were incubated with phosphate-buffered saline instead of antibodies. After mounting with resinene, the sections were evaluated under high magnification (100× and 400×).

### Western blot

Protein samples were extracted from left ventricular myocardium using Protein Extraction Kit (ZSGB Biotechnology Co., Ltd., Beijing, China). The protein concentrations were determined using the bicinchoninic acid protein assay (Beyotime Biotechnology, Jiangsu, China). Equal amounts (50 μg) of protein were subjected to 10% SDS-PAGE, transferred to a polyvinylidene difluoride membrane, blocked with 5% non-fat milk in Tris-buffered saline (TBS; 1 h at room temperature), and incubated overnight at 4 °C with a primary antibody: anti-p-Cx43 (1:300, SC-101660, Santa Cruz Biotechnology, Santa Cruz, CA, USA), anti-t-Cx43 (1:300, BA1727, Boster Biological Technology Co., Ltd., California, USA), anti-G-CSFR (1:300, SC-9173, Santa Cruz Biotechnology, Santa Cruz, CA, USA), anti-t-JAK2 (1:300, SC-278, Santa Cruz Biotechnology, Santa Cruz, CA, USA), anti-p-JAK2 (1:300, SC-21870, Santa Cruz Biotechnology, Santa Cruz, CA, USA), anti-t-STAT3 (1:300, SC-8019, Santa Cruz Biotechnology, Santa Cruz, CA, USA), anti-p-STAT3 (1:300, SC-8059, Santa Cruz Biotechnology, Santa Cruz, CA, USA), or anti-β-actin (1:1000, Cell Signaling Technology, Danvers, MA, USA). β-actin was used as internal control. After three washes in TBS-Tween-20, the membranes were incubated with the appropriate secondary antibody labeled with HRP (1:3000, ZSGB Biotechnology Co., Ltd., Beijing, China) in the dark for 1 h at room temperature. Protein bands were developed by electrochemiluminescence, and their intensities were analyzed using the ImageJ software (National Institutes of Health, Bethesda, MD, USA).

### Statistical analysis

Data are presented as means ± standard deviations and were analyzed using SPSS 19.0 (IBM Corp., Armonk, NY, USA). Normally distributed data (according to the Kolmogorov-Smirnoff test) with homogeneity of variance were compared between groups using one-way analysis of variance (ANOVA) and the least significant difference (LSD) test. Data within each group between baseline and 2 weeks were tested using the paired t-test. Data that were not normally distributed or had heterogeneity of variance were analyzed using the Kruskal-Wallis U test. VT/VF rate and mortality rate were compared using the chi-square test. *P* < 0.05 was considered statistically significant. Prism 5 (Graphpad, La Jolla, CA, USA) was used for graph plotting.

## Results

### Rabbit body weight declined after surgery in the CME group but not in the other groups

**Fig. 1:**
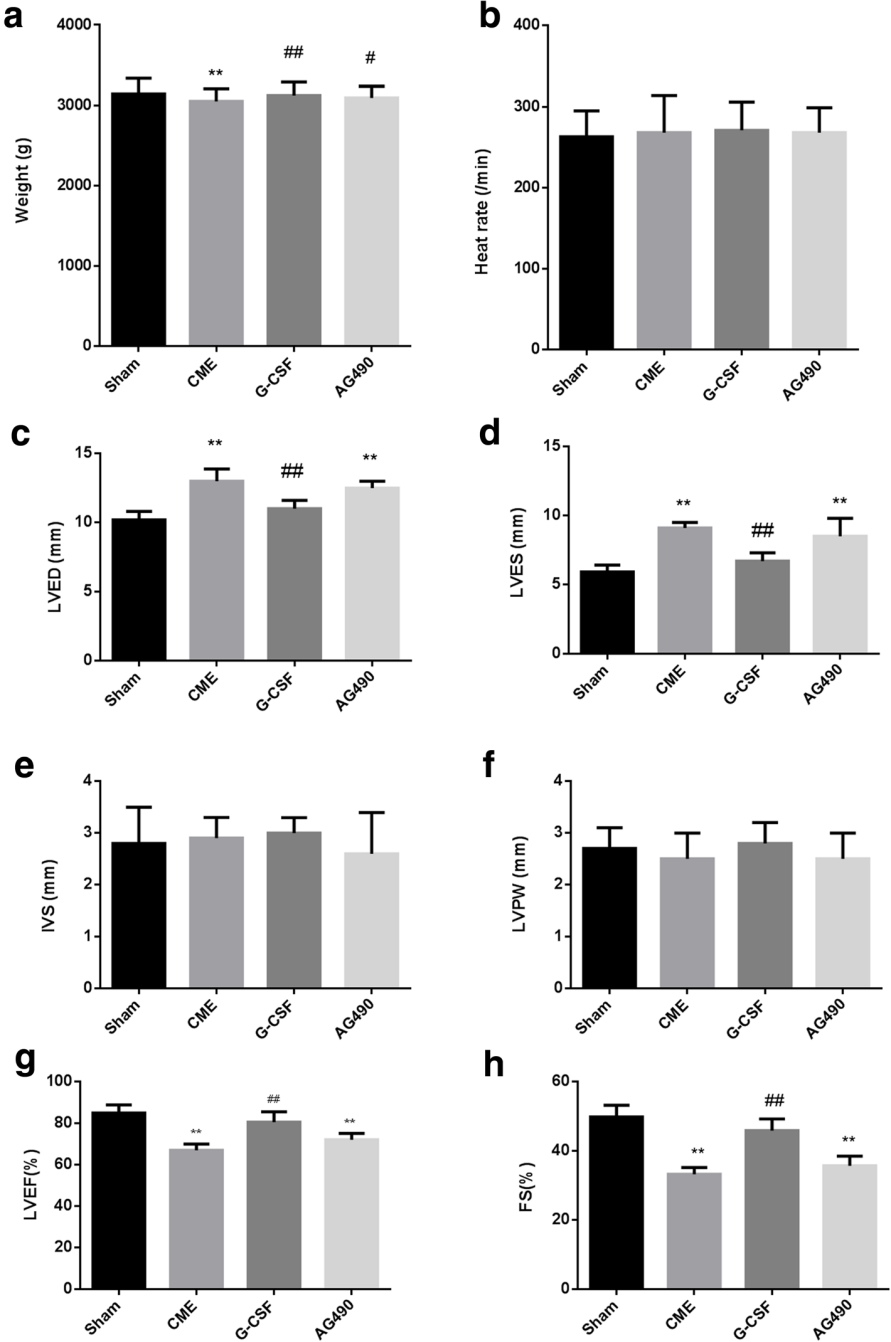
Bodyweight, heart rate, and echocardiography parameters for each group after surgery. **a** Bodyweight. **b** Heart rate. **c** Parasternal left ventricular end-diastolic dimension (LVED). **d** Left ventricular end-systolic dimension (LVES). **e** Interventricular septum dimension (IVS). **f** Left ventricle posterior wall dimension (LVPW). **g** Left ventricular ejection fraction (LVEF). **h** Fractional shortening (FS). Data are shown as the means ± standard deviations (*n* = 10/group, except *n* = 9 in the AG490 group). *P < 0.05, **P < 0.01 vs. Sham group; ##*P* < 0.01 vs. CME group (one-way ANOVA with LSD post hoc test)

One rabbit from the AG490 group died during the experiments, and 39 rabbits completed the study. Before surgery, there were no significant differences in body weight among groups. Bodyweight at 2 weeks after surgery was significantly lower in the CME group than in the Sham (*P* < 0.01), G-CSF (*P* < 0.01), and AG490 (*P* < 0.05) groups (Fig. 1a). There were no significant differences in body weight among the Sham, G-CSF, and AG490 groups at 2 weeks after surgery (Fig. 1a). Heart rate did not differ significantly among groups after surgery (Fig. 1b).

### Echocardiography parameters changed after surgery in the CME and AG490 groups but not in the sham and G-CSF groups

Before surgery, there were no significant differences among the groups in any of the echocardiography parameters (Fig. 1c–h). At 2 weeks after surgery, the CME group had a significantly higher LVED and LVES (*P* < 0.01) and a significantly lower LVEF and FS (*P* < 0.01) than the Sham and G-CSF groups (Fig. 1c, d, g, h). Moreover, LVED, LVES, LVEF, and FS exhibited no significant differences between the G-CSF and Sham groups or between the CME and AG490 groups (Fig. 1c, d, g, h), indicating that G-CSF alleviated the effects of CME and that AG490 blocked the effects of G-CSF. IVS and LVPW were similar among the four groups (Fig. 1E, F).

### CME-induced susceptibility to ventricular arrhythmia was attenuated by G-CSF via the JAK2-STAT3 pathway

**Fig. 2:**
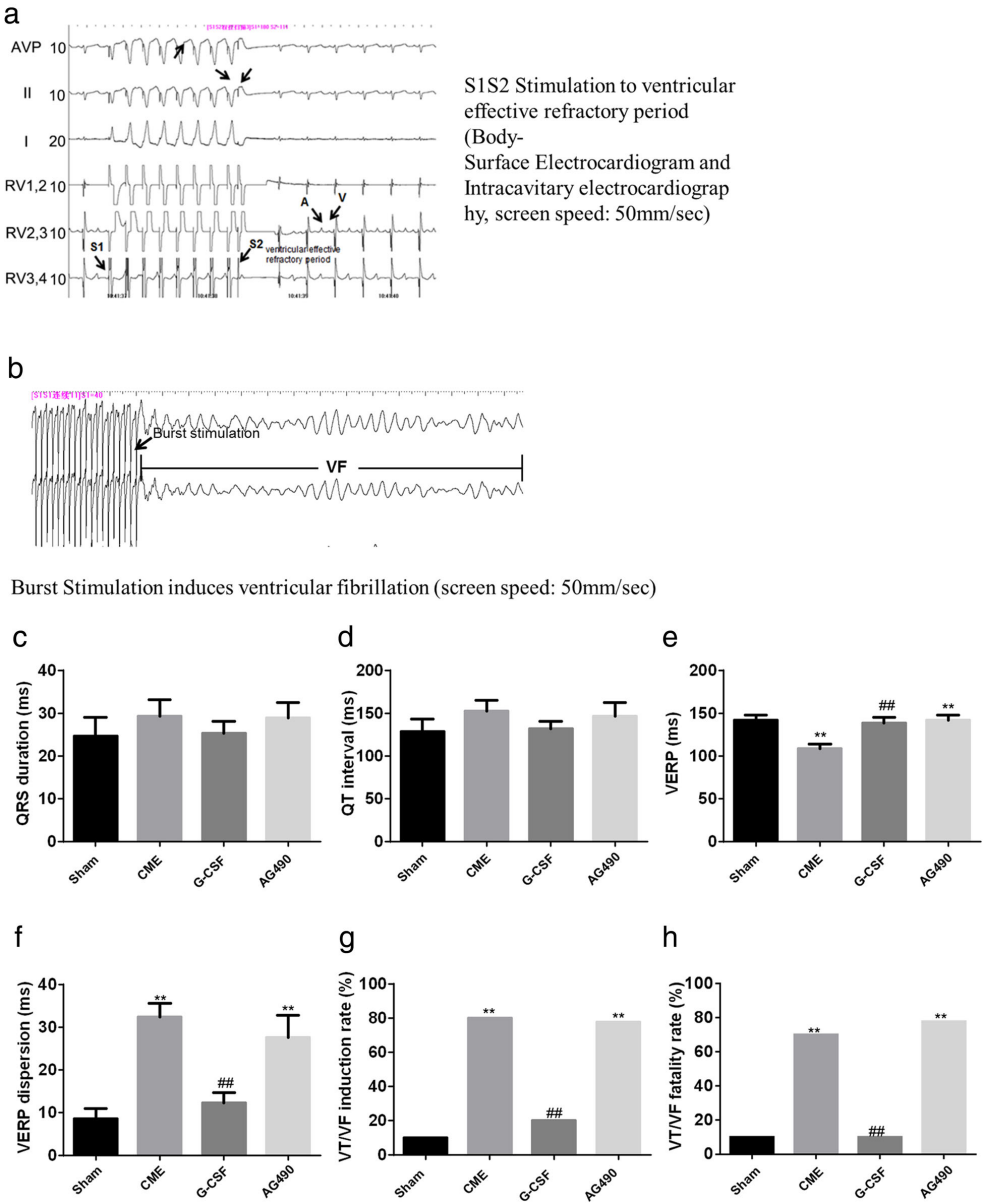
Results of electrophysiology tests performed 2 weeks after surgery. **a** Representative electrophysiology traces (surface electrocardiogram and intracavitary electrocardiography) showing S1S2 stimulation to determine the ventricular effective refractory period (VERP). Screen speed: 50 mm/s. **b** Representative electrophysiology trace demonstrating ventricular fibrillation induced by burst stimulation. **c** QRS duration. **d** QT interval. **e** VERP. **f** VERP dispersion. **g** Ventricular tachycardia or ventricular fibrillation (VT/VF) induction rate. **h** VT/VF fatality rate. **c** – **f** Data are shown as means ± standard deviations (*n* = 10/group, except *n* = 9 in the AG490 group). **P < 0.01 vs. Sham group; ##*P* < 0.01 vs. CME group (one-way ANOVA with LSD post hoc test). **g**-**h**, Data are shown as percentage, **P < 0.01 vs. Sham group; ##*P* < 0.01 vs. CME group (Chi-square test)

Representative electrophysiology data are shown in Fig. 2a–b. QRS duration and QT interval did not differ significantly between groups (Fig. 2c, d). VERP was significantly shorter in the CME group than in the Sham and G-CSF groups (*P* < 0.01) and significantly shorter in the AG490 group than in the Sham group (*P* < 0.01) (Fig. 2e). Notably, there were no significant differences in VERP between the Sham and G-CSF groups or the CME and AG490 groups (Fig. 2e), suggesting that G-CSF alleviated the effects of CME and that AG490 blocked the effects of G-CSF. VERP dispersion was significantly higher in the CME and AG490 groups as compared with the Sham and G-CSF groups (*P* < 0.01), with no significant differences between the Sham and G-CSF groups or the CME and AG490 groups (Fig. 2f). Similarly, the VT/VF induction rate and VT/VF mortality rate were significantly higher in the CME and AG490 groups than in the Sham and G-CSF groups (*P* < 0.01), with no significant differences between the former two groups or latter two groups (Fig. 2g, h). Taken together, the results suggest that G-CSF restored the changes to be similar to those of the Sham group, while AG-490 (a JAK2 inhibitor) blocked the effects of G-CSF.

### G-CSF reduces infarct size and interstitial collagen fiber deposition in the myocardium via the JAK2-STAT3 pathway

**Fig. 3:**
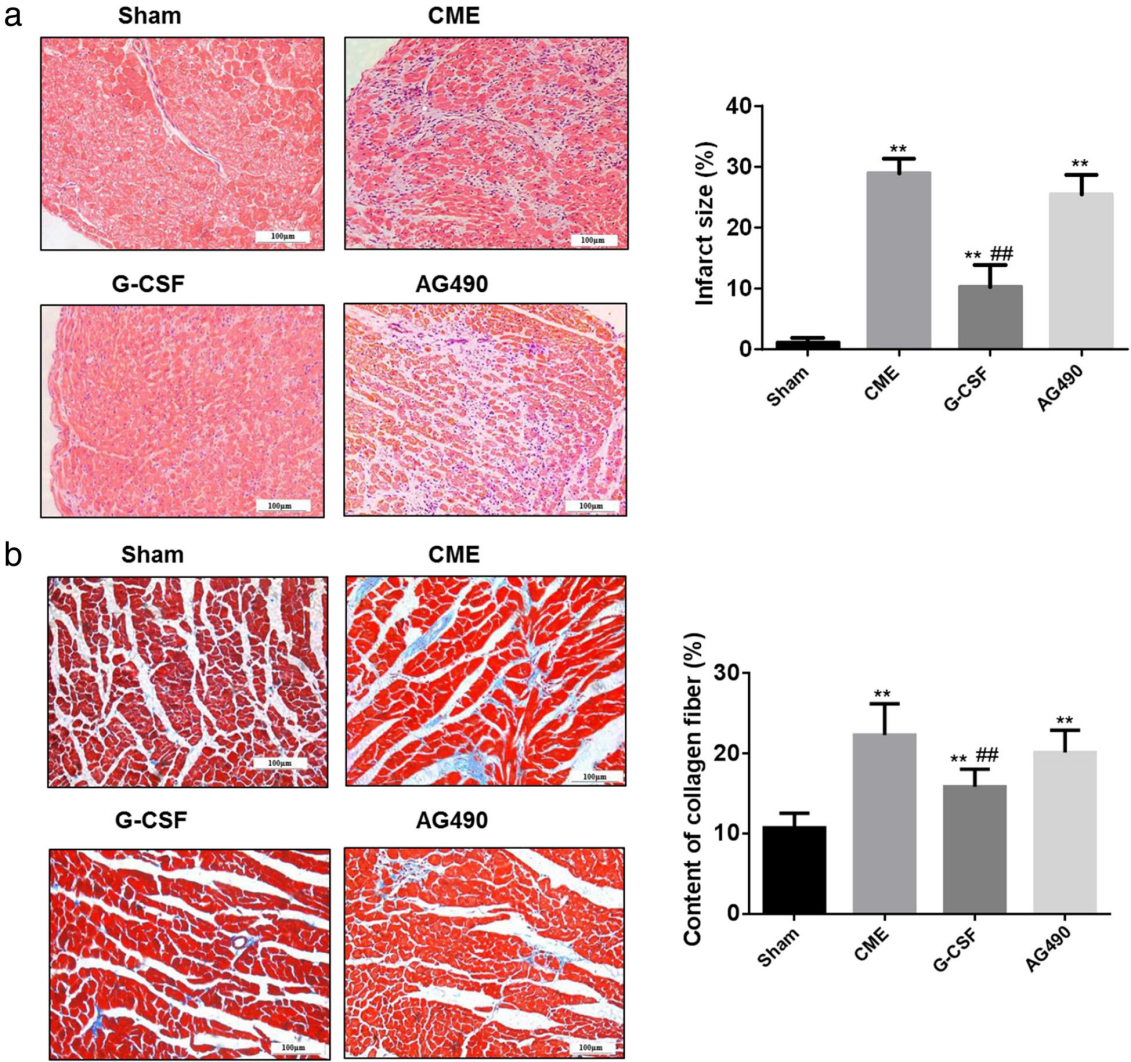
Myocardial infarct size and interstitial collagen fiber content evaluated 2 weeks after surgery. **a** Images of ventricular myocardium stained with hematoxylin-eosin to show the regions of infarcted myocardium (200×). In the Sham group, the cytoplasm of the rabbit ventricular myocytes was uniformly stained pink, and the nuclei were stained blue-black. In the CME group, the infarcted areas were clearly evident around the arterioles, with edema, degeneration of peripheral myocardium, and infiltration of peripheral inflammatory cells. Compared with the CME group, the infarcted region was much smaller in the G-CSF group but similar in size in the AG490 group. Quantification of the size of the microinfarcted region as a proportion of the total area is shown in the graph on the right. Data are shown as means ± standard deviations of at least three independent experiments. **P < 0.01 vs. Sham group; ##*P* < 0.01 vs. CME group (one-way ANOVA with LSD post hoc test). **b** Images of ventricular myocardium stained with Masson trichrome to show the interstitial collagen fiber content (200×). The cardiomyocytes are stained red, and collagen is stained blue. Interstitial collagen content was notably higher in the CME, G-CSF, and AG490 groups than in the Sham group. The interstitial deposition of collagen was not as great in the G-CSF group as in the CME and AG490 groups. Interstitial collagen content (excluding collagen around blood vessels) as a proportion of the total area is quantified in the graph on the right. Data are shown as means ± standard deviations (*n* = 10/group, except *n* = 9 in the AG490 group). **P < 0.01 vs. Sham group; ##*P* < 0.01 vs. CME group (one-way ANOVA with LSD post-hoc test)

HE-stained sections of the myocardium demonstrated a normal arrangement of myocardial cells in the Sham group. The CME and AG490 groups exhibited multiple circular regions of microinfarction with inflammatory cell infiltration, but these abnormalities were less prominent in the G-CSF group (Fig. 3a). The microinfarcted area was significantly larger in the CME, G-CSF, and AG490 groups than in the Sham group (*P* < 0.01), but significantly smaller in the G-CSF group than in the CME or AG490 groups (*P* < 0.01; Fig. 3a). Staining with Masson trichrome revealed that interstitial collagen deposition was significantly increased in the CME, G-CSF, and AG490 groups compared with the Sham group (*P* < 0.01), but interstitial collagen content was significantly lower in the G-CSF group than in the CME or AG490 groups (*P* < 0.01; Fig. 3b). Again, G-CSF alleviated the effects of CME, and that AG490 (a JAK2 inhibitor) blocked the effects of G-CSF.

### G-CSF increases Cx43 phosphorylation and G-CSFR expression via the JAK2-STAT3 pathway

**Fig. 4:**
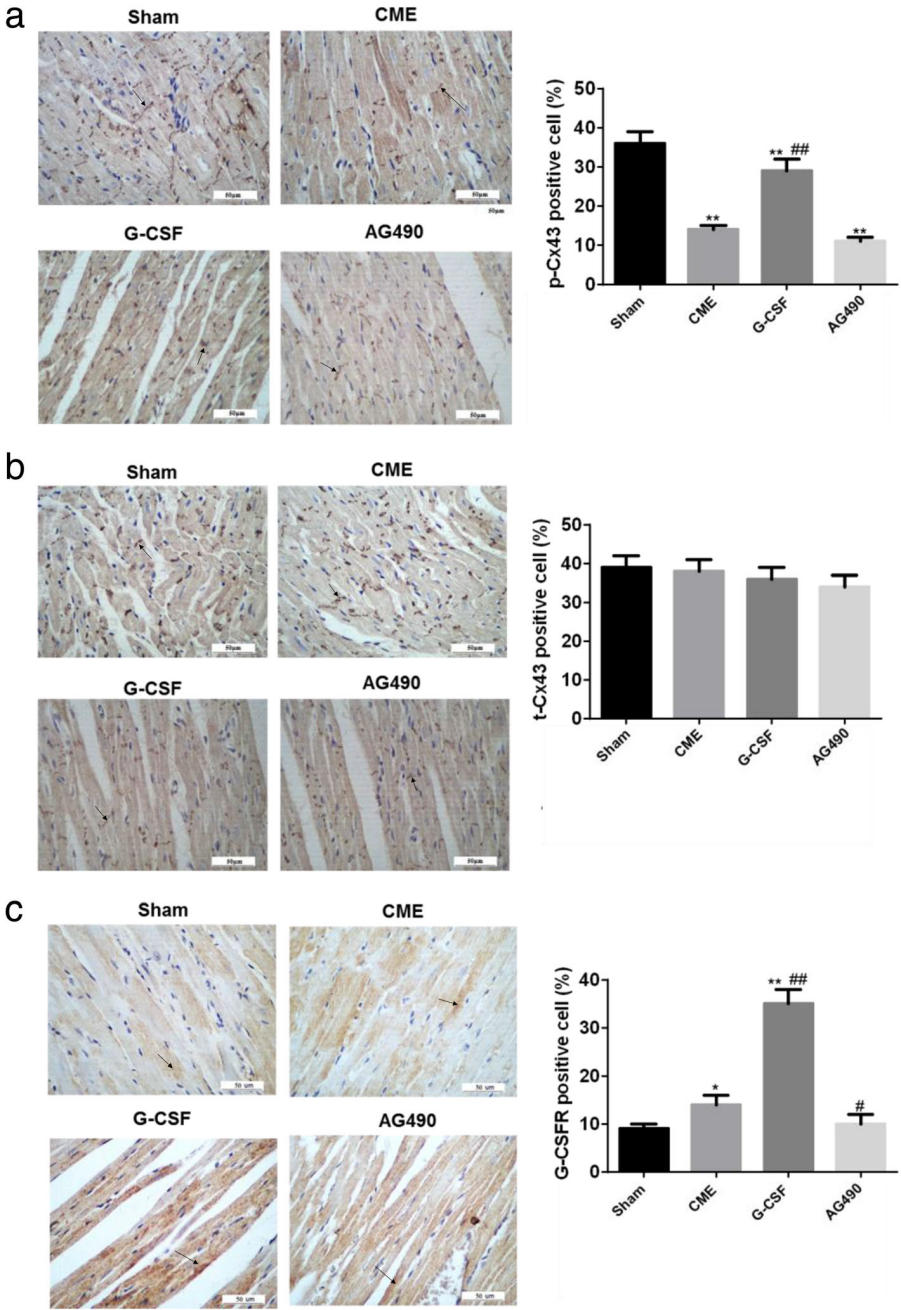
Expressions of p-Cx43, t-Cx43, and G-CSFR proteins in rabbit myocardium from each group. The expression levels of each protein were determined using immunohistochemistry. Positive staining is shown in brown; the nuclei are stained blue-black. **a** p-Cx43. **b** t-Cx43. **c** G-CSFR. Quantification of expression was performed under high magnification (400×). The black arrows indicate representative enriched areas, but not all areas are indicated by arrows in the images. Data are shown as means ± standard deviations (*n* = 10/group, except *n* = 9 in the AG490 group). *P < 0.05, **P < 0.01 vs. Sham group; #*P* < 0.05, ##*P* < 0.01 vs. CME group (one-way ANOVA with LSD post-hoc test)

Immunohistochemistry showed that in the Sham group, the p-Cx43 protein was distributed mainly at the longitudinal junctions between cells where intercalated discs are located (Fig. 4a). By contrast, p-Cx43 showed a disordered distribution in the CME and AG490 groups, with substantial lateralization of p-Cx43 and only limited expression at the longitudinal junctions between myocytes (Fig. 4a). The distribution pattern in the G-CSF group was between that of the Sham group and the two other groups (Fig. 4a). The p-Cx43 expression level was significantly lower in the Sham group than in the other groups (*P* < 0.01) and substantially higher in the G-CSF group than in the CME or AG490 groups (*P* < 0.01) (Fig. 4a). The cellular localization of t-Cx43 in each group was similar to that of p-Cx43 (Fig. 4b). There were no significant differences among groups in the t-Cx43 expression levels (Fig. 4b). G-CSFR expression was much higher in the G-CSF group than in the other groups (*P* < 0.01; Fig. 4c). G-CSFR expression was slightly higher in the CME group than in the Sham and AG490 groups (*P* < 0.05; Fig. 4c). Those results suggest that G-CSF alleviated the changes in Cx43 expression induced by CME and that AG490 (a JAK2 inhibitor) blocked the effects of G-CSF.

**Fig. 5:**
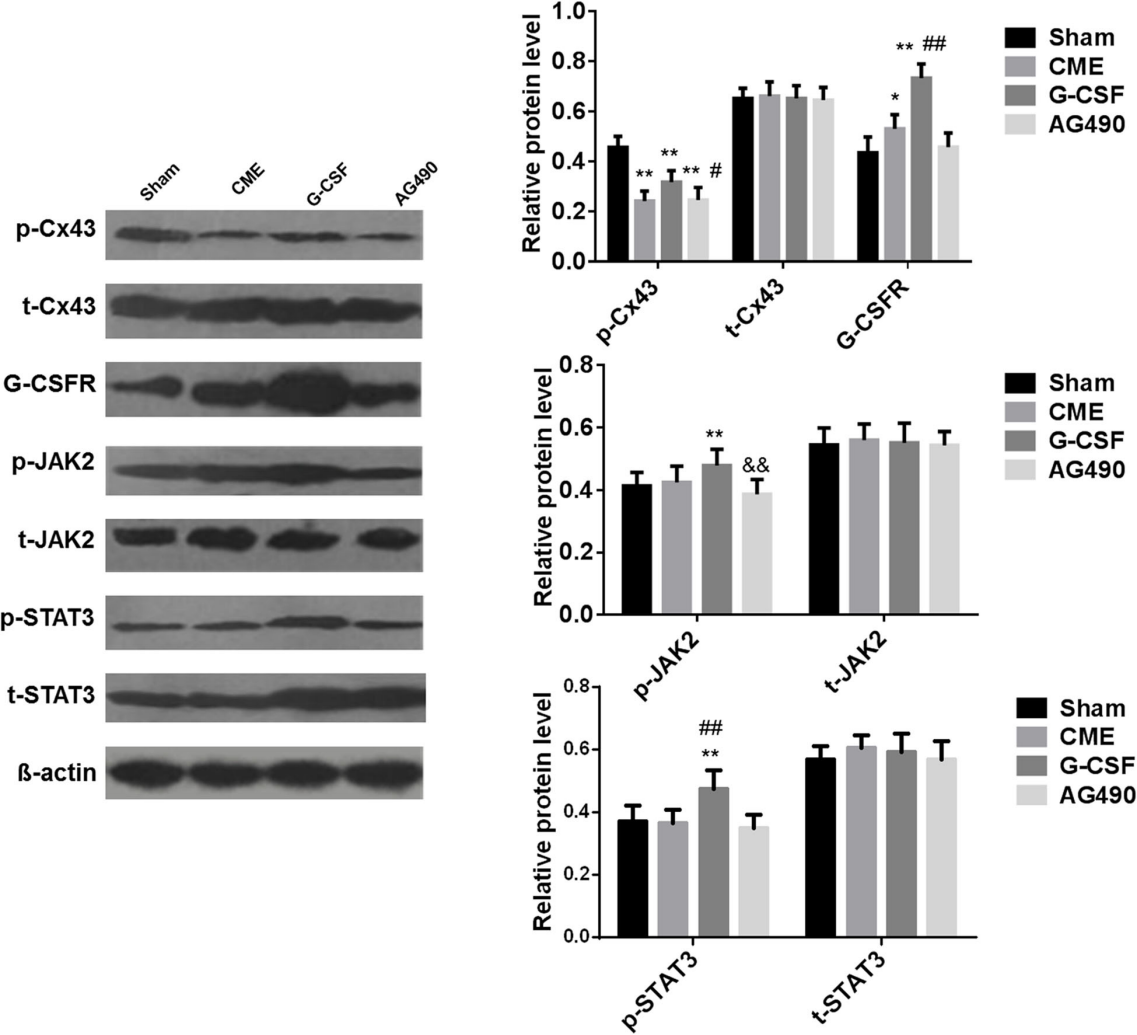
Expression levels of the p-Cx43, t-Cx43, G-CSFR, p-JAK2, t-JAK2, p-STAT3, and t-STAT3 proteins in rabbit myocardium from each group. The expression levels of each protein were determined using western blot. Left: representative immunoblots showing the expression of the various proteins in each group. Right: quantification of the protein bands. Data are shown as means ± standard deviations (*n* = 10/group, except *n* = 9 in the AG490 group). *P < 0.05, **P < 0.01 vs. Sham group; #*P* < 0.05, ##*P* < 0.01 vs. CME group; && *P* < 0.01 vs. G-CSF group (one-way ANOVA with LSD post-hoc test)

Western blot experiments were performed to measure the protein expression levels of p-Cx43, t-Cx43, G-CSFR, p-JAK2, t-JAK2, p-STAT3, and t-STAT3 (Fig. 5). The western blot data for p-Cx43, t-Cx43, and G-CSFR were consistent with the immunohistochemistry results. There were no significant differences among groups in the expression levels of the t-JAK2 and t-STAT3 proteins (Fig. 5). The expression levels of p-JAK2 and p-STAT3 were significantly higher in the G-CSF group than in the Sham group (*P* < 0.01), but these elevations were attenuated in the AG490 group (Fig. 5).

## Discussion

An innovation of the present study is the description of a new rabbit model of CME, which was established by selective injection of autologous microthrombus into the LAD artery through a catheter inserted under the guidance of digital subtraction angiography. A notable finding of the present study was that CME in rabbits resulted in decreased cardiac function (reductions in LVEF and FS and increases in LVED and LVES) and electrical remodeling (increases in VERP, VERP dispersion, susceptibility to VT/VF, and mortality of VT/VF) that were accompanied by reduced levels of p-Cx43 and a redistribution of Cx43. Importantly, G-CSF partially or completely reversed these effects of CME, and the beneficial actions of G-CSF were attenuated by AG490, an inhibitor of JAK2-STAT3 signaling. Taken together, our findings suggest that G-CSF might act via the JAK2-STAT3 pathway to inhibit structural and electrical remodeling of the heart and enhanced susceptibility to ventricular arrhythmia following CME. This finding is another innovation of the present study.

Most previous preclinical studies of CME used rat models induced by an autologous thrombus to simulate the rupture of a plaque in vivo [18–20], but the rat model of CME has several shortcomings. First, a thoracotomy is necessary for establishment of the model, which may lead to inflammation that affects the coronary microcirculation [21]. Second, selective coronary artery branch embolization cannot be achieved, making it difficult to control the extent of embolization [22]. Third, electrophysiology assessments such as measurement of VERP dispersion are challenging in rats due to the small ventricular volume [23]. A rabbit model of acute MI has been reported using selective embolization of the anterior descending branch via auricular artery catheterization [24], and this model has the advantages of being minimally invasive while mimicking the pathophysiological process of acute MI. In the present study, we described a new rabbit model of CME, which was established by selective injection of autologous microthrombus into the LAD artery through a catheter inserted under the guidance of digital subtraction angiography. Our pilot experiments established that injection of 5 mg of autologous thromboembolic particles caused distal occlusion of small coronary arteries similar to that seen in patients who experience CME from a ruptured coronary atherosclerotic plaque. Furthermore, our rabbit model of CME minimized trauma to the animal and was associated with a low mortality rate. Thus, we believe that our novel rabbit model is well suited to the study of CME.

Although MI and myocardial microinfarction may share some pathophysiological features [25], the mechanisms underlying structural and electrical remodeling of the heart after CME remain incompletely understood. A notable finding of our study was that CME led to increases in VERP and VERP dispersion as well as enhanced susceptibility to VT/VF and a higher rate of VT/VF-associated mortality. VERP dispersion has long been considered a mechanism underlying the initiation of reentrant arrhythmias (28). In addition to electrical remodeling, we also observed reduced Cx43 expression and abnormal Cx43 distribution in the heart after CME. In addition to its role in intercellular electrical communication, Cx43 has been proposed to regulate voltage-gated sodium current (29). Abnormal Cx43 expression has been reported to cause conduction slowing [26] and contribute to the electrical remodeling after MI that results in abnormal coordination of electrical activity and arrhythmogenesis [27]. Decreased Cx43 phosphorylation and an alteration in Cx43 distribution from intercalated discs to lateral regions of cardiomyocytes are thought to result in electrical conduction disorders and arrhythmia [28, 29]. Furthermore, overexpression of Cx43 in the myocardium was shown to improve the conduction velocity in the infarct margin and reduce the susceptibility to ventricular arrhythmia [30]. Therefore, the electrical remodeling after CME in our study was likely due, at least in part, to reduced levels of p-Cx43 and lateralization of Cx43.

G-CSF has received increasing attention as a cytokine that promotes repair after MI [8]. G-CSF has been shown to accelerate angiogenesis and reduce fibrosis in a swine model of cardiac ischemia/reperfusion [31]. The present study revealed that the G-CSF administration was able to inhibit CME-induced myocardial electrical remodeling, prolongation of VERP, increased VERP dispersion, and enhanced susceptibility to ventricular arrhythmias. Our findings are in agreement with previous reports that G-CSF stabilized cardiac electrophysiological characteristics after ischemia-reperfusion injury or MI, shortened the duration of the cardiac action potential, prolonged the effective refractory period and reduced the susceptibility to ventricular arrhythmia [9, 10].

The mechanisms by which G-CSF exerts the above effects are unclear. Previous investigations reported that G-CSF causes bone marrow cells to migrate to infarcted regions and differentiate into cardiomyocytes and stimulates monocytes and neutrophils to promote neovascularization through paracrine mechanisms [32]. On the other hand, not all studies implicated bone marrow cells in the effects of G-CSF after MI [33]. Notably, G-CSF has been reported to improve cardiac function and promote cardiomyocyte survival after MI via a mechanism involving JAK2-STAT3 signaling [34]. Moreover, G-CSF was shown to attenuate myocardial apoptosis following CME in rats. Here, we provide evidence for the first time that G-CSF inhibited abnormal Cx43 expression and electrical remodeling after CME by activating JAK2-STAT3 signaling. Consistent with our findings, activation of JAK2-STAT3 signaling was found to induce Cx43 expression in astrocytes [35], and G-CSF was shown to decrease the incidence of ventricular arrhythmias by increasing Cx43 expression in the infarct margin and shortening the action potential duration [36]. We propose that G-CSF acts via the G-CSFR to activate JAK2-STAT3 signaling, promote the expression of Cx43, attenuate myocardial electrical remodeling, and reduce the susceptibility to ventricular arrhythmias after CME.

The present study provides novel insight into CME, which affects 10–30% of patients and seriously affecting their prognosis [2]. No drugs or mechanical devices are currently available to prevent CME, and remedial measures after the occurrence of CME have only a limited impact on the development of arrhythmias and prognosis [5]. Therefore, the present study provides novel insight into CME, which affects 10–30% of patients and seriously affecting their prognosis [2]. The results suggest that the use of G-CSF could be a promising way to improve the prognosis of those patients through the modulation of the JAK2-STAT3 pathway, structural remodeling, and electrical remodeling.

This study has some limitations. First, when generating the CME model, extraction of the microcatheter is challenging, and air embolism is more likely in rabbits than in larger animals because of narrow coronary arteries. Second, the patch-clamp technique was not used to directly measure the action potential duration of myocytes in the infarct site and peripheral zone, and the roles of ion channels were not investigated. Third, only AG490 was used [35, 37–39], but other JAK inhibitors are available, and the results should be confirmed using additional inhibitors. Finally, among all structural proteins of the heart, only Cx43 was selected, and only G-CSF was tested. Additional studies are needed to clarify the mechanism of electrical remodeling after CME.

## Conclusions

In conclusion, our findings suggest that G-CSF acts via the JAK2-STAT3 pathway to inhibit the abnormal Cx43 expression, electrical remodeling, and enhanced susceptibility to ventricular arrhythmias that occur after CME.

## Data Availability

The study data are available from the corresponding author upon reasonable request.

## Abbreviations

CME: Coronary microembolization
Cx43: Connexin-43
G-CSF: Granulocyte colony-stimulating factor
G-CSFR: G-CSF receptor
LAD: Left anterior descending
PCI: Percutaneous coronary intervention
p-Cx43: Phosphorylated Cx43
VERP: Ventricular effective refractory period
VF: Ventricular fibrillation
VT: Ventricular tachycardia

## References

[1] Gaffar R, Habib B, Filion KB, Reynier P, Eisenberg MJ. Optimal Timing of Complete Revascularization in Acute Coronary Syndrome: A Systematic Review and Meta-Analysis. J Am Heart Assoc. 2017;6(4):e005381. 10.1161/JAHA.116.005381 PMC5533029 28396570

[2] Lim SY (2016). No-reflow Phoenomenon by intracoronary Thrombus in acute myocardial infarction. Chonnam Med J.

[3] Zhang Y, Zhang L, Zheng H, Chen H (2018). Effects of atrial fibrillation on complications and prognosis of patients receiving emergency PCI after acute myocardial infarction. Exp Ther Med.

[4] Ma J, Qian J, Chang S, Chen Z, Jin H, Zeng M (2014). Left ventricular remodeling with preserved function after coronary microembolization: the effect of methylprednisolone. Eur J Med Res.

[5] Salinas P, Jimenez-Valero S, Moreno R, Sanchez-Recalde A, Galeote G, Calvo L (2012). Update in pharmacological management of coronary no-reflow phenomenon. Cardiovasc Hematol Agents Med Chem.

[6] Solan JL, Lampe PD (2018). Spatio-temporal regulation of connexin43 phosphorylation and gap junction dynamics. Biochim Biophys Acta Biomembr.

[7] Kessler EL, Boulaksil M, van Rijen HV, Vos MA, van Veen TA (2014). Passive ventricular remodeling in cardiac disease: focus on heterogeneity. Front Physiol.

[8] D'Amario D, Leone AM, Borovac JA, Cannata F, Siracusano A, Niccoli G (2018). Granulocyte colony-stimulating factor for the treatment of cardiovascular diseases: an update with a critical appraisal. Pharmacol Res.

[9] Liu HM, Luo T, Zhou X, Cai L, Huang TG, Jiang TM (2011). Disassociation between left ventricular mechanical and electrical properties in ischemic rat heart after G-CSF treatment. Cardiovasc Drugs Ther.

[10] Kanlop N, Thommasorn S, Palee S, Weerateerangkul P, Suwansirikul S, Chattipakorn S (2011). Granulocyte colony-stimulating factor stabilizes cardiac electrophysiology and decreases infarct size during cardiac ischaemic/reperfusion in swine. Acta Physiol (Oxf).

[11] Eid RA, Alkhateeb MA, Eleawa S, Al-Hashem FH, Al-Shraim M, El-Kott AF (2018). Cardioprotective effect of ghrelin against myocardial infarction-induced left ventricular injury via inhibition of SOCS3 and activation of JAK2/STAT3 signaling. Basic Res Cardiol.

[12] Tang Y, Tong X, Li Y, Jiang G, Yu M, Chen Y (2018). JAK2/STAT3 pathway is involved in the protective effects of epidermal growth factor receptor activation against cerebral ischemia/reperfusion injury in rats. Neurosci Lett.

[13] Li H, Spagnol G, Zheng L, Stauch KL, Sorgen PL (2016). Regulation of Connexin43 function and expression by tyrosine kinase 2. J Biol Chem.

[14] Zhao Q, Sun C, Xu X, Zhou J, Wu Y, Tian Y (2013). Early use of granulocyte colony stimulating factor improves survival in a rabbit model of chronic myocardial ischemia. J Cardiol.

[15] Chen G, Wu J, Sun C, Qi M, Hang C, Gong Y (2008). Potential role of JAK2 in cerebral vasospasm after experimental subarachnoid hemorrhage. Brain Res.

[16] Liu L, Mu Y, Han W, Wang C (2014). Association of hypercholesterolemia and cardiac function evaluated by speckle tracking echocardiography in a rabbit model. Lipids Health Dis.

[17] Krishnamoorthi S, Perotti LE, Borgstrom NP, Ajijola OA, Frid A, Ponnaluri AV (2014). Simulation methods and validation criteria for modeling cardiac ventricular electrophysiology. PLoS One.

[18] Liang J, Li L, Sun Y, He W, Wang X, Su Q (2017). The protective effect of activating Nrf2 / HO-1 signaling pathway on cardiomyocyte apoptosis after coronary microembolization in rats. BMC Cardiovasc Disord.

[19] Su Q, Li L, Sun Y, Yang H, Ye Z, Zhao J (2018). Effects of the TLR4/Myd88/NF-kappaB signaling pathway on NLRP3 Inflammasome in coronary microembolization-induced myocardial injury. Cell Physiol Biochem.

[20] Su Q, Lv X, Sun Y, Ye Z, Kong B, Qin Z (2018). Role of TLR4/MyD88/NF-kappaB signaling pathway in coronary microembolization-induced myocardial injury prevented and treated with nicorandil. Biomed Pharmacother.

[21] Zhu H, Ding Y, Xu X, Li M, Fang Y, Gao B (2017). Prostaglandin E1 protects coronary microvascular function via the glycogen synthase kinase 3beta-mitochondrial permeability transition pore pathway in rat hearts subjected to sodium laurate-induced coronary microembolization. Am J Transl Res.

[22] Fu FY, Chen BY, Chen LL, Zhang FL, Luo YK, Jun F (2016). Improvement of the survival and therapeutic effects of implanted mesenchymal stem cells in a rat model of coronary microembolization by rosuvastatin treatment. Eur Rev Med Pharmacol Sci.

[23] Bere Z, Obrenovitch TP, Kozak G, Bari F, Farkas E (2014). Imaging reveals the focal area of spreading depolarizations and a variety of hemodynamic responses in a rat microembolic stroke model. J Cereb Blood Flow Metab.

[24] Katsanos K, Mitsos S, Koletsis E, Bravou V, Karnabatidis D, Kolonitsiou F (2012). Transauricular embolization of the rabbit coronary artery for experimental myocardial infarction: comparison of a minimally invasive closed-chest model with open-chest surgery. J Cardiothorac Surg.

[25] Heusch G, Skyschally A, Kleinbongard P (2018). Coronary microembolization and microvascular dysfunction. Int J Cardiol.

[26] Fontes MS, van Veen TA, de Bakker JM, van Rijen HV (2012). Functional consequences of abnormal Cx43 expression in the heart. Biochim Biophys Acta.

[27] Leo-Macias A, Agullo-Pascual E, Delmar M (2016). The cardiac connexome: non-canonical functions of connexin43 and their role in cardiac arrhythmias. Semin Cell Dev Biol.

[28] Boengler K, Schulz R (2017). Connexin 43 and mitochondria in cardiovascular health and disease. Adv Exp Med Biol.

[29] Duffy HS (2012). The molecular mechanisms of gap junction remodeling. Heart Rhythm.

[30] Greener ID, Sasano T, Wan X, Igarashi T, Strom M, Rosenbaum DS (2012). Connexin43 gene transfer reduces ventricular tachycardia susceptibility after myocardial infarction. J Am Coll Cardiol.

[31] Sato T, Suzuki H, Kusuyama T, Omori Y, Soda T, Tsunoda F (2008). G-CSF after myocardial infarction accelerates angiogenesis and reduces fibrosis in swine. Int J Cardiol.

[32] Klocke R, Kuhlmann MT, Scobioala S, Schabitz WR, Nikol S (2008). Granulocyte colony-stimulating factor (G-CSF) for cardio- and cerebrovascular regenerative applications. Curr Med Chem.

[33] Sato D, Otani H, Fujita M, Shimazu T, Yoshioka K, Enoki C (2012). Granulocyte colony-stimulating factor does not enhance recruitment of bone marrow-derived cells in rats with acute myocardial infarction. Exp Clin Cardiol.

[34] Harada M, Qin Y, Takano H, Minamino T, Zou Y, Toko H (2005). G-CSF prevents cardiac remodeling after myocardial infarction by activating the Jak-stat pathway in cardiomyocytes. Nat Med.

[35] Ozog MA, Bernier SM, Bates DC, Chatterjee B, Lo CW, Naus CC (2004). The complex of ciliary neurotrophic factor-ciliary neurotrophic factor receptor alpha up-regulates connexin43 and intercellular coupling in astrocytes via the Janus tyrosine kinase/signal transducer and activator of transcription pathway. Mol Biol Cell.

[36] Kuhlmann MT, Kirchhof P, Klocke R, Hasib L, Stypmann J, Fabritz L (2006). G-CSF/SCF reduces inducible arrhythmias in the infarcted heart potentially via increased connexin43 expression and arteriogenesis. J Exp Med.

[37] Seo IA, Lee HK, Shin YK, Lee SH, Seo SY, Park JW (2009). Janus kinase 2 inhibitor AG490 inhibits the STAT3 signaling pathway by suppressing protein translation of gp130. Korean J Physiol Pharmacol.

[38] Kimura K, Nishida T (2010). Role of the ubiquitin-proteasome pathway in downregulation of the gap-junction protein Connexin43 by TNF-{alpha} in human corneal fibroblasts. Invest Ophthalmol Vis Sci.

[39] Wang J, Bai T, Wang N, Li H, Guo X (2020). Neuroprotective potential of imatinib in global ischemia-reperfusion-induced cerebral injury: possible role of Janus-activated kinase 2/signal transducer and activator of transcription 3 and connexin 43. Korean J Physiol Pharmacol.

